# Five-year results of iontophoresis-assisted transepithelial corneal cross-linking for keratoconus

**DOI:** 10.1007/s10792-023-02768-1

**Published:** 2023-07-03

**Authors:** Anna Maria Roszkowska, Giovanni William Oliverio, Katarzyna Hydzik-Sajak, Michele De Crescenzo, Pasquale Aragona

**Affiliations:** 1 Ophthalmology Clinic, Department of Biomedical Sciences, University of Messina, Messina, Italy; 2 Ophthalmology Clinic, Faculty of Medicine and Health Sciences, Andrzej Frycz Modrzewski Kraków University, Kraków, Poland

**Keywords:** Keratoconus, Transpithelial corneal cross-linking, Iontophoresis

## Abstract

**Purpose:**

To assess long-term efficacy and safety of iontophoresis-assisted transepithelial corneal cross-linking (I-CXL) for keratoconus.

**Patients and methods:**

Twenty-seven eyes of 21 patients (15 M, 6F) affected by progressive keratoconus were evaluated. All subjects were treated with iontophoresis-assisted transepithelial CXL. The patients were examined at baseline and each 6 months after the CXL procedure. Only subjects who completed the follow-up of 5 years were considered in this study. The main outcome measures were uncorrected visual acuity (UCVA), corrected visual acuity (CDVA), corneal transparency and corneal parameters such as K-max, central corneal thickness (CCT) and at the thinnest point, and high-order ocular aberrations (HOAs). The ABCD system was used to determine the progression and re-progression of ectasia.

**Setting:**

Ophthalmology Clinic, University Hospital of Messina, Messina, Italy.

**Results:**

At 5 years, significant improvements of UCVA from 0.53 ± 0.33 logMAR to 0.4 ± 0.33 logMAR (*p* = 0.001) and HOAs (*p* = 0.01) were registered. No significant changes of CDVA (*p* = 0.4), K-max (*p* = 0.75), CCT (*p* = 0.5) were observed at the end of follow-up period.

The ABCD system showed re-progression in 25.9% of eyes after 5 years. No adverse events such as corneal opacities and infections were reported.

**Conclusions:**

Iontophoresis-assisted transepithelial CXL resulted to be safe and effective to stabilize progressive keratoconus in adults at a long-term follow-up.

## Introduction

Keratoconus (KC) is an ectatic corneal disease, characterized by progressive corneal thinning and steepening associated with a variable visual impairment [1, 2].

Although it was commonly defined as a non-inflammatory disease, the recent scientific evidences confirmed the etiopathogenic role of inflammatory cytokines and matrix metalloproteinases [2, 3].

KC manifests predominantly at puberty and progresses with different rate till the fourth decade of life when usually stops [4, 5].

The corneal collagen cross-linking (CXL) procedure slows down or halts the progression of corneal ectasia, improved stromal rigidity, due to formation of the covalent bonds between collagen fibrils in the stroma as a result of photochemical reaction between riboflavin and ultraviolet A light (UV-A) [6, 7].

Although the CXL standard protocol proved to be effective to halt and stabilize KC with the observational period of up to 15 years, it may present some limitations associated to the epithelial debridement and postoperative discomfort [8–11].

On the other hand, the transepithelial cross-linking technique (TE-CXL), proposed to improve safety and to reduce the postoperative discomfort and complications due to the epithelial debridement, is demonstrated to be ineffective, particularly in the young patients [12–14]. The in vitro studies on human corneas showed a reduced penetration of riboflavin into the corneal stroma with epithelium on corneal soaking [12–14].

The introduction of iontophoresis to enhance the penetration of riboflavin permitted to soak the corneal stroma with preserved epithelial layer making the procedure less invasive and shortening significantly the duration of the procedure [15]. Indeed, several studies demonstrated that iontophoresis-assisted transepithelial corneal imbibition resulted in greater and deeper riboflavin saturation than in the TE-CXL technique, but less effective if compared to standard protocol [15–20].

Although iontophoresis-assisted epi-on CXL presents numerous advantages, there are only few long-term studies that evaluated its efficacy to halt KC progression.

The aim of this study is to assess long-term efficacy and safety of iontophoresis-assisted transepithelial corneal cross-linking (I-CXL) for keratoconus**.**

## Methods

In this retrospective study were evaluated patients with progressive keratoconus undergoing iontophoresis-assisted epi-on CXL and completed the 5-year follow-up.

Inclusion criteria were: documented diagnosis of evaluative keratoconus over the twelve months preceding treatment, age between 18 and 40 years, and no systemic diseases.

Exclusion criteria were: a minimum corneal thickness < 400 μm, a maximum K steeper than 61 D, corneal scarring, previous ocular surgery, and other ocular diseases.

All procedures were performed at the Ophthalmology Unit of the University Hospital of Messina. This study was approved by the Institutional Review Board of the University of Messina (Protocol number 45/18) and was conducted accordingly to the tenets of the Declaration of Helsinki.

The examination included visual acuity assessment with both uncorrected distance visual acuity (UDVA) and corrected distance visual acuity (CDVA) using logMAR chart, slit lamp examination to evaluate corneal transparency, corneal tomography using Pentacam HR® (Oculus, Inc., Germany), specular microscopy for endothelial cell count (Konan SP8000, Keeler, Japan), Goldmann applanation tonometry and ophthalmoscopy.

The tomographic data were used to assess the corneal parameters and comprised flattest keratometric reading (K-flat), steepest keratometric reading (K-steep), maximum keratometry value (K-max), minimum corneal thickness (MCT), central corneal thickness (CCT). Total high-order aberrations root main square (HOA RMS) was registered with 6 mm diameter zone. For each parameter, the mean of three consecutive measurements was considered for evaluation. The cone location was determined on tangential algorithm maps; central keratoconus was considered when the K-max was located within the central 3 mm, while paracentral keratoconus was diagnosed with K-max located between 3 and 5 mm from the center.

The main outcome measures were evaluated at baseline and after 5 years after the treatment.

The ABCD progression of ectasia was evaluated, considering a variation for each parameter more than 95% CI to the follow-up 12 months after surgery, to determine the progression of keratoconus after treatment.

### I-CXL procedure

The procedure was conducted under topical anesthesia with 0.2% oxybuprocaine hydrochloride 0.4% (Novesine, Laboratoires Thea, France) disposable eye drops after 2% pilocarpine drops instillation to induce miosis necessary for reducing the UV-A intraocular penetration.

The iontophoresis device (Iontofor-CXL, Fidia Farmaceutici S.p.A., Italy), used to soak the cornea with riboflavin, comprises two electrodes connected to an electric power generator. The positive electrode contained in a patch was applied on the forehead of the patient, while the negative electrode, formed by a stainless steel grid inserted in a corneal applicator with an annular suction ring, was placed on the corneal surface of the patient and maintained with a vacuum system. The applicator was filled with a negatively charged solution of hypo-osmolar dextran-free 0.1% riboflavin (Ricrolin Plus, Fidia Farmaceutici S.p.A Italy), and then a continuous current generator set at 1.0 mA induced the corneal stroma soaking for 5 min.

The ultraviolet device (VEGA CBM-X-Linker, CSO, Firenze, Italy) with a wavelength of 370 nm set at 10 mW/cm^2^ and at a distance of 45 mm was used for the corneal irradiation for 9 min to achieve a total irradiance of 5.4 J/cm^2^ (Table 1). The overall treatment duration was of 14 min. After CXL a soft contact lens was applied. All patients received the same postoperative topical treatment with antibiotic drops (Ofloxacin 3 mg/ml, Fidia Farmaceutici S.p.A) 4 times daily for 5 days, and lubricating eye drops with hyaluronic acid, amino acids and vitamin B2 (Ribolisin, Fidia Farmaceutici S.p.A, Italy) were used 4 times daily, for 6 months.

**Table 1:** Corneal collagen cross-linking method

Parameter	I-ON CXL
Fluence total (J/cm^2^)	5.4
Soak time (min)	5
Intensity (mW)	10
Treatment time (min)	9
Epithelium status	On
Chromophore	Riboflavin (Ricrolin Plus)
Chromophore osmolarity	hypo-osmolar dextran-free
Chromophore concentration	0.1%
Light source	VEGA CBM-X-Linker
Irradiation mode (interval)	Continuous

*J* Joule; *W* Watt; *Min* minute

### Statistical analysis

The numerical data were expressed as mean and standard deviation and the categorical variables as absolute frequency and percentage. The normal distribution was confirmed by the Kolmogorov–Smirnov test for all parameters.

For each parameter, in order to evaluate the level of statistical significance in different time points of observation, the ANOVA for repeated measurements and correct *p*-values with Bonferroni correction were applied.

Additionally, univariate and multivariate regression analyses were performed to evaluate the association between KC progression after treatment and preoperative parameters such as corneal pachymetry at thinnest point, KC location, and K-max.*p*-values less than 0.05 were considered statistically significant. The STATA 15.1 software (Stata, Inc., College Station, TX) was used.

## Results

Twenty-seven eyes of 21 patients (15 males and 6 females) with the age range from 22 to 32 years (mean age 25.72 ± 2.86 years) were evaluated.

The mean UCVA showed statistically significant change at 5 years (*p* = 0.001), whereas the CDVA was unvaried throughout the follow-up (*p* = 0.4) (Table 2).

**Table 2:** Ionophoresis corneal cross-linking group at baseline and 1, 2, 3, 4 and 5 years after treatment

Parameter	Baseline	1 year	2 years	3 years	4 years	5 years	*p*-value
UCVA, logMAR	0.50 ± 0.33	0.45 ± 0.36	0.44 ± 0.35	0.42 ± 0.35	0.4 ± 0.33	0.38 ± 0.32	**0.001**
CDVA, logMAR	0.16 ± 0.23	0.13 ± 0.18	0.13 ± 0.22	0.13 ± 0.22	0.13 ± 0.22	0.13 ± 0.21	0.4
Sphere, D	0.86 ± 1.26	1.1 ± 1.6	1.02 ± 1.7	1.0 ± 1.6	1.2 ± 1.8	1.3 ± 1.61	0.18
Cylinder, D	2.35 ± 1.60	2.56 ± 1.52	2.83 ± 1.49	2.75 ± 1.51	2.7 ± 1.52	2.55 ± 1.48	0.17
MRSE, D	2.13 ± 1.19	2.26 ± 1.81	2.28 ± 1.82	2.22 ± 1.9	2.34 ± 1.94	2.49 ± 1.71	0.07
K-max, D	57.88 ± 6.61	57.74 ± 7.14	57.81 ± 6.81	57.86 ± 7.02	57.88 ± 7.1	57.96 ± 7.16	0.75
K-flat, D	46.53 ± 4.14	46.63 ± 4.48	46.52 ± 4.45	46.63 ± 4.42	46.51 ± 4.44	46.51 ± 4.41	0.2
K-steep, D	49.74 ± 4.43	50.18 ± 4.59	50.26 ± 4.59	50.24 ± 4.56	50.2 ± 4.52	50.28 ± 4.47	0.07
HOAs, µm	3.25 ± 1.17	3.02 ± 1.1	3.11 ± 1.15	3.12 ± 1.29	3.05 ± 1.3	2.89 ± 1.35	**0.01**
Thinnest point, µm	467.5 ± 51.1	465.1 ± 50.1	46.3 ± 49.8	462.7 ± 50	462.2 50.9	463.2 ± 49.32	0.06
CCT, µm	484.4 ± 50.74	481.5 ± 49.53	482 ± 50.54	481.9 ± 49.7	483 ± 50.3	484.8 ± 48.26	0.5
ECD	2458 ± 184.8	2388 ± 151	2371 ± 144.9	2378 ± 146.7	2369 ± 148.1	2378 ± 142.6	0.21

*UCVA* uncorrected visual acuity; *CDVA* corrected distance visual acuity; *LogMAR* logarithm of the minimum angle of resolution; *K-max* maximum keratometry value; *D* diopters; *HOAs* high-order aberrations; *CCT* central corneal thickness; *μm* micrometers; *ECD* endothelial cells count. All data are reported as mean ± standard deviation and median. Bold character for *p* < 0.05

At the end of follow-up, the mean K-max, K-flat and K-steep did not reveal significant changes (*p* = 0.75, *p* = 0.2 and *p* = 0.07respectively), but in 4 eyes (18.5%) an increment of K-max > 1D was detected.

Furthermore, at 5 years no statistically significant changes of pachymetry at the thinnest point (*p* = 0.06) and CCT (*p* = 0.5) were observed.

No significant changes in refractive parameters were observed, whereas HOAs demonstrated a significant reduction (*p* = 0.01) (Table 1).

Endothelial cell count changes and corneal haze based on slit lamp examination were not registered throughout the follow-up period (Table 2).

No intraoperative or postoperative complications occurred in this series of patients.

Considering the ABCD progression system, after 5 years 7 eyes (25.9%) presented a progression of KC and were recommended to retreatment with accelerated Epi-OFF CXL (intensity: 9 mW; treatment time: 9 min; fluence 5.4 J/cm^2^).

### Factors associated to progression of keratoconus after I-CXL

Fourteen eyes presented a central KC location, whereas 13 eyes presented a paracentral location. Twelve eyes treated were right, whereas 13 were left eyes.

In multiple regression analysis, baseline pachymetry at the thinnest point (*p* = 0.66), K-max (*p* = 0.47), age of treatment (*p* = 0.28), eye laterality (*p* = 0.74) and KC location (*p* = 0.79) did not correlate with re-progression of KC. No patients presented atopic diseases.

## Discussion

I-CXL represents an effective technique to enhance the penetration of riboflavin through the epithelium and soaking the corneal stroma [15–18].

In vitro studies on human corneas, treated with I-CXL, assessed the biomechanical effect, riboflavin diffusion and distribution, and demonstrated that iontophoresis procedure increases significantly the stromal penetration of riboflavin as compared to the TE-CXL [15–17]. Additionally, previous clinical studies confirmed the effectiveness of I-CXL in short-medium-term follow-up, reporting good clinical results in halting the keratoconus progression [18].

Indeed, several studies reported improvement of UCVA and CDVA after 12 and 24 months, as well as a decrease in HOAs. Moreover, stabilization and regression of keratometry values were reported at 1 and 2 years of follow-up [18–20].

When compared to S-CXL, I-CXL reached a lower stromal concentration of riboflavin, and some clinical studies reported differences on stabilization of progressive keratoconus [15–17, 19, 20].

Cantemir et al., in a randomized controlled clinical trial compared I-CXL to S-CXL, reported similar results in the two groups, and confirming the non-inferiority of I-CXL for stopping the progression of keratoconus at 3 years [21].

Furthermore, in a recent meta-analysis on 723 eyes, comparing both techniques, no conclusive evidences were found for either topographic or visual acuity outcomes at 12 months or later after surgery [22].

However, there are numerous studies that demonstrated the efficacy of S-CXL to arrest the progression of keratoconus with a long-term follow-up, for I-CXL few data are available [23, 24].

Wu et al. reported 5 years results of TE-CXL with two continuous cycles of iontophoresis (a variation of the manufacturer’s recommended technique), demonstrating its efficacy in stabilization of progressive keratoconus, and a low rate of progression after treatment (11.6% of patients) [25].

In a recent study, Vinciguerra et al. reported results up to 7 years in a small cohort of patients (5 patients completed the follow-up), and their findings demonstrated that I-CXL allowed to halt KC also in a long-term period [26]. The authors reported a progression of KC in 26.3% of patients using the new ABCD progression system [26]. Our results confirm such outcome in greater number of subjects observed at 5-year follow-up.

The K-max variation is still widely considered as progression evaluation parameter and used to report CXL outcome; nevertheless, there is a wide variability on K-max measurements that could invalidate these findings [26, 27]. In our study, although the mean K-max did not changed after 5 years (*p* = 0.72), and 23 eyes (81.5%) did not demonstrate changes of K-max values, in 4 eyes (18.5%) an increment of K-max > 1D was detected.

Hence considering actual progression criteria based on K-max worsening, the 81.5% of our patients were stable after 5 years from the I-CXL procedure.

Recently, Belin et al. proposed a novel classification for KC, the ABCD system with 95% CI of the CXL population, that allows to determining accurately progression in ectatic corneas [28–30].

However, this new evaluation procedure is not widely used yet, probably because related to the diagnostic platform, actually it is not possible to compare the data based only on K-max variation with ABCD progression system.

In a long-term study on S-CXL, there was a progression rate of keratoconus after CXL of 7.6%, and high preoperative K-max values and the presence of atopic disease emerged as the main risk factors for progression of keratoconus after CXL [31].

Indeed, in a recent retrospective study on 230 eyes treated with epi-off CXL, preoperative K-max value and neurodermatitis combined with other atopic diseases were significant risk factors for new progression of keratoconus after CXL [32].

In accordance with Vinciguerra et al., considering the ABCD progression of ectasia system, at 5 years in our group 7 patients (25.9%) presented a KC progression [26].

This finding confirms the less efficacy of I-CXL compared to S-CXL, to halt keratoconus progression in a long-term period.

In accordance with previous studies, our findings revealed significant improvement of UCVA and HOAs at the end of follow-up with no significant changes of CDVA and topographic data [18, 20, 26].

This study presents some limitations: first of all, the retrospective design and relatively small sample size. Moreover, our findings did not demonstrate significant morphological and keratometry improvements after treatment, as well as did not include particular cases such as thin corneas (< 400 μm) or pediatric patients (< 18 years old).

However, for the best of our knowledge this is the first study that reports 5-year follow-up in a larger cohort of patients treated with I-CXL.

In conclusion, our findings, in accordance with previous studies, confirmed the capacity of I-CXL to halt KC, without evident adverse events, with a 25.9% of KC progression rate in the long-term follow-up, with limited improvement of keratometry values and functional parameters.

However, additional studies with a larger sample size are necessary to assess the long-term results and effectiveness of I-CXL using ABCD evaluation system.
